# Plasma proteomic analysis reveals key pathways associated with divergent residual body weight gain phenotype in beef steers

**DOI:** 10.3389/fvets.2024.1415594

**Published:** 2024-07-22

**Authors:** Modoluwamu Idowu, Godstime Taiwo, Taylor Sidney, Anjola Adewoye, Ibukun M. Ogunade

**Affiliations:** ^1^Division of Animal Science, West Virginia University, Morgantown, WV, United States; ^2^Department of Chemistry, West Virginia University, Morgantown, WV, United States

**Keywords:** feed efficiency, proteome, metabolic process, cattle, immune signaling

## Abstract

We utilized plasma proteomics profiling to explore metabolic pathways and key proteins associated with divergent residual body weight gain (RADG) phenotype in crossbred (Angus × Hereford) beef steers. A group of 108 crossbred growing beef steers (average BW = 282.87 ± 30 kg; age = 253 ± 28 days) were fed a high-forage total mixed ration for 49 days in five dry lot pens (20–22 beef steers per pen), each equipped with two GrowSafe8000 intake nodes to determine their RADG phenotype. After RADG identification, blood samples were collected from the beef steers with the highest RADG (most efficient; *n* = 15; 0.76 kg/d) and lowest RADG (least efficient; *n* = 15; −0.65 kg/d). Plasma proteomics analysis was conducted on all plasma samples using a nano LC–MS/MS platform. Proteins with FC ≥ 1.2 and false-discovery rate-adjusted *p-*values (FDR) ≤ 0.05 were considered significantly differentially abundant. The analysis identified 435 proteins, with 59 differentially abundant proteins (DAPs) between positive and negative-RADG beef steers. Plasma abundance of 38 proteins, such as macrophage stimulating 1 and peptidase D was upregulated (FC ≥ 1.2, FDR ≤ 0.05) in positive-RADG beef steers, while 21 proteins, including fibronectin and ALB protein were greater (FC < 1.2, FDR ≤ 0.05) in negative-RADG beef steers. The results of the Gene Ontology (GO) analysis of all the DAPs showed enrichment of pathways such as metabolic processes, biological regulation, and catalytic activity in positive-RADG beef steers. Results of the EuKaryotic Orthologous Groups (KOG) analysis revealed increased abundance of DAPs involved in energy production and conversion, amino acid transport and metabolism, and lipid transport and metabolism in positive-RADG beef steers. The results of this study revealed key metabolic pathways and proteins associated with divergent RADG phenotype in beef cattle which give more insight into the biological basis of feed efficiency in crossbred beef cattle.

## Introduction

In animal production, the efficient utilization of feed resources is of paramount concern, mainly due to the high cost of feed (1, 2). To address these challenges, the adoption of phenotypic and genetic selection strategies centered around feed efficiency measurements has become increasingly prevalent. In beef cattle, two widely used indicators of feed efficiency are Residual feed intake (RFI) and Residual body weight gain (RADG). Residual feed intake defined by Koch et al. (2) and Herd et al. (3), quantifies the difference between an animal’s actual and expected dry matter intake (DMI), considering its production and maintenance needs. This involves a regression analysis that considers DMI, body weight (BW), and body weight gain (4). Identification of animals with higher feed efficiency, characterized by negative RFI values, indicates they consume less feed than expected, while positive RFI values signify less efficient animals consuming more feed than expected. Residual body weight gain, following a similar principle as RFI, focuses on the rate of body weight gain, regressed against feed intake and body weight (5). Positive-RADG values denote desirable feed-efficient animals with enhanced body weight gain, while negative values highlight less efficient animals in terms of growth at the same level of DMI (5). Improved RADG can lead to faster growth rates and potentially shorter time to market, which can have significant economic benefits for producers. It is important to note that RADG, like RFI, is influenced by genetic and environmental factors; however, RADG may offer more robust insights in varied environmental conditions by reflecting how growth responds to these conditions.

Over the past decade, high-throughput omics technologies such as proteomics, transcriptomics, and metabolomics have been applied to identify genes, proteins, and biological pathways associated with divergent selection for RFI (6). Proteomics techniques allow for the quantification of protein abundance under different physiological conditions or in response to various stimuli to better understand how protein abundance levels fluctuate during different metabolic states and how animals adapt to changes in their environment or nutritional status (7, 8). Some studies have harnessed proteomic approaches to uncover alterations in protein profiles within blood and skeletal muscle of ruminants (9–11). For instance, Carvalho et al. (10) revealed significant differences in protein abundance within the skeletal muscle of Nellore beef cattle with divergent RFI, suggesting variations in energy expenditure as a contributing factor to observed feed efficiency differences. Additionally, Elolimy et al. (11) indicated that the most feed-efficient beef cattle exhibited a greater abundance of critical proteins involved in cellular protein synthesis. While previous research has focused on critical pathways and proteins influencing RFI, the proteome profiles of beef cattle with divergent RADG phenotype have not been investigated. Our study employed plasma proteomics profiling to investigate metabolic pathways and key proteins associated with divergent RADG phenotype in crossbred beef steers. We hypothesized that beef steers with divergent RADG phenotype would exhibit variations in plasma proteome profiles.

## Materials and methods

### Animals, feeding, and RADG determination

The research procedures were approved by the Institutional Animal Care and Use Committees of West Virginia (protocol number 2204052569). A group of 108 crossbred (Angus × Hereford) growing beef steers (average BW = 282.87 ± 30 kg; age = 253 ± 28 days) were fed a high-forage total mixed ration (TMR), primarily consisting of corn silage; ground hay; and a ration balancing supplement; CP = 13.2%, NDF = 45.9%, and NEg = 0.93 Mcal/kg; calculated based on NASEM (12). The animals were kept in five dry lot pens (20–22 beef steers per pen) each equipped with two GrowSafe8000 intake nodes (GrowSafe Systems Ltd., Airdrie, Alberta, Canada) for a period of 49 d to determine their RADG phenotype. Details of animal feeding and procedures have been reported in our previous study (13). Briefly, the beef steers were allowed to adapt to the feeding facilities and diet for 15 days. Following this period, individual feed intake was measured for 49 days. Daily BW for each animal, measured using In-Pen Weighing Positions (IPW, Vytelle LLC), was regressed on time using simple linear regression to calculate initial BW, mid-test BW, and average daily gain (ADG). The initial BW, mid-test metabolic BW, and ADG were calculated by regressing the daily BW for each animal using simple linear regression. The ADG of each beef steer was regressed against their daily DMI and mid-test metabolic BW (MMTW = mid-test BW^0.75^), and the RADG was calculated as the difference between the predicted value of the regression and the actual measured value based on the following equation: Y = β0 + β_1_X_1_ + β_2_X_2_ + ε, where Y is the ADG (kg/d), β_0_ is the regression intercept, β_1_ and β_2_ are the partial regression coefficients, X_1_ is the MMTW (kg), X_2_ is the observed DMI (kg/d) (2, 5). At the end of the feeding trial, the beef steers were ranked based on their RADG coefficients. The most efficient beef steers with the greatest positive-RADG (*n* = 15) and the least efficient beef steers with the least negative-RADG (*n* = 15) were identified.

### Blood collection and plasma preparation

Upon completion of the feeding trial, blood samples were collected from both positive-RADG beef steers (*n* = 15) and negative-RADG beef steers (*n* = 15) via the coccygeal vessel. These samples were collected using 10-mL Vacutainer tubes containing sodium heparin (Vacutainer, Becton Dickinson, Franklin Lakes, NJ, United States). Post-collection, the blood samples were promptly placed on ice and subsequently centrifuged at 2,500 × *g* for 15 min at 4°C to separate the plasma. The separated plasma samples were then stored at −80°C until further analysis could be conducted.

### Protein identification and quantification

The plasma samples underwent a rigorous processing procedure to prepare them for identification and quantification using a nano LC–MS/MS platform (14, 15). The plasma samples were first depleted of high-abundant proteins, such as albumin and immunoglobulin G, using a depletion spin-column procedure (16). The depleted plasma samples were digested with trypsin, and subsequently identified, and quantified by applying the nano LC–MS/MS platform.

The depletion spin column was equilibrated to room temperature, and 10 μL of the sample was added directly to the resin slurry in the column. After capping the column, it was gently mixed to ensure complete suspension of the resin in the solution and underwent a 30-min incubation at room temperature with gentle end-over-end mixing. Following incubation, the column was centrifuged to remove the resin, leaving behind a filtrate containing the sample with removed albumin and IgG. To further refine the sample, ice acetone was added, and the mixture was incubated overnight at −20°C. After centrifugation, the supernatant was discarded, and the remaining acetone was removed. The samples were then dissolved in 50 mM ammonium bicarbonate and centrifuged. Subsequently, the concentrated samples underwent reduction and alkylation before trypsin digestion. Finally, the extracted peptides were lyophilized and resuspended in 20 μL of 0.1% formic acid before LC–MS/MS analysis (17).

### Nano LC–MS/MS analysis

For the nano-liquid chromatography (NLC) phase of the analysis, we employed the Nanoflow UPLC: Ultimate 3000 nano UHPLC system from ThermoFisher Scientific, USA. This system achieves high-resolution separations for subsequent mass spectrometry analysis. The nanocolumn setup included a trapping column (PepMap C18, 100 μm × 2 cm, 5 μm) and an analytical column (PepMap C18, 100 Å, 75 μm × 50 cm, 2 μm). A sample volume of 1 μg was loaded onto the system, and the mobile phase consisted of two components: A, which contained 0.1% formic acid in water, and B, which comprised 0.1% formic acid in 80% acetonitrile. The total flow rate was maintained at 250 nL/min. A full scan was conducted over the m/z range of 300–1,650 at a resolution of 60,000 at 200 m/z. The automatic gain control target for the full scan was set at 3e^6^, ensuring accurate and reliable mass measurements. Additionally, the MS/MS scan operated in Top 20 mode with settings such as a resolution of 15,000 at 200 m/z, an automatic gain control target of 1e^5^, a maximum injection time of 19 ms, and a normalized collision energy of 28%.

### Statistical and bioinformatics analysis

We processed the thirty raw MS files by searching against the *Bos taurus* protein database using Maxquant (version 1.6.2.6). Parameters included carbamidomethylation (C) (fixed) and oxidation (M) (variable) for protein modifications, trypsin enzyme specificity, and a maximum of 2 missed cleavages. Precursor ion mass tolerance was set to 10 ppm, with an MS/MS tolerance of 0.5 Da. We visualized proteome differences between positive-RADG and negative-RADG beef steers using partial least squares discriminant analysis (PLS-DA) scores plot and identified DAPs through volcano plot analysis (FC ≥ 1.2, FDR-adjusted *p*-values ≤0.05). Further analysis included GO enrichment and KOG classification of the identified proteome.

### GO enrichment analysis

The proteomic dataset’s gene ontology annotations were sourced from the UniProt-GOA database.^1^ Protein IDs were initially converted to UniProt IDs and subsequently linked to GO IDs. The proteins were then categorized based on their GO annotations into three groups: biological process, cellular component, and molecular function. Enrichment analysis was conducted using Fisher’s exact test to compare differentially abundant proteins to all identified proteins within each category. GO terms with a false discovery rate (FDR) ≤ 0.05 were deemed statistically significant.

## Results

The results of the growth performance of the beef steers with divergent RADG phenotype are shown in Table 1. The average RADG values of positive-RADG and negative-RADG beef steers were 0.76 and −0.65 kg/d, respectively. The initial BW and DMI were similar (*p* > 0.05) for the two groups; however, final body weight and ADG were greater (*p* = 0.01) in beef steers with positive-RADG (1.25 kg/d) than those with negative-RADG (0.94 kg/d).

**Table 1:** Growth performance of beef steers with divergent residual body weight gain phenotype.

Item	Positive-RADG	Negative-RADG	SEM	*p*-value
RADG (kg/d)	0.76	−0.65	0.10	0.01
Initial weight (kg)	273	286	11.9	0.31
Final weight (kg)	345	338	1.83	0.01
ADG (kg/d)	1.29	0.94	0.09	0.01
DMI (kg/d)	7.39	7.94	0.49	0.27

SEM, standard error of the mean; ADG, average daily gain; DMI, dry matter intake.

**Figure 1:**
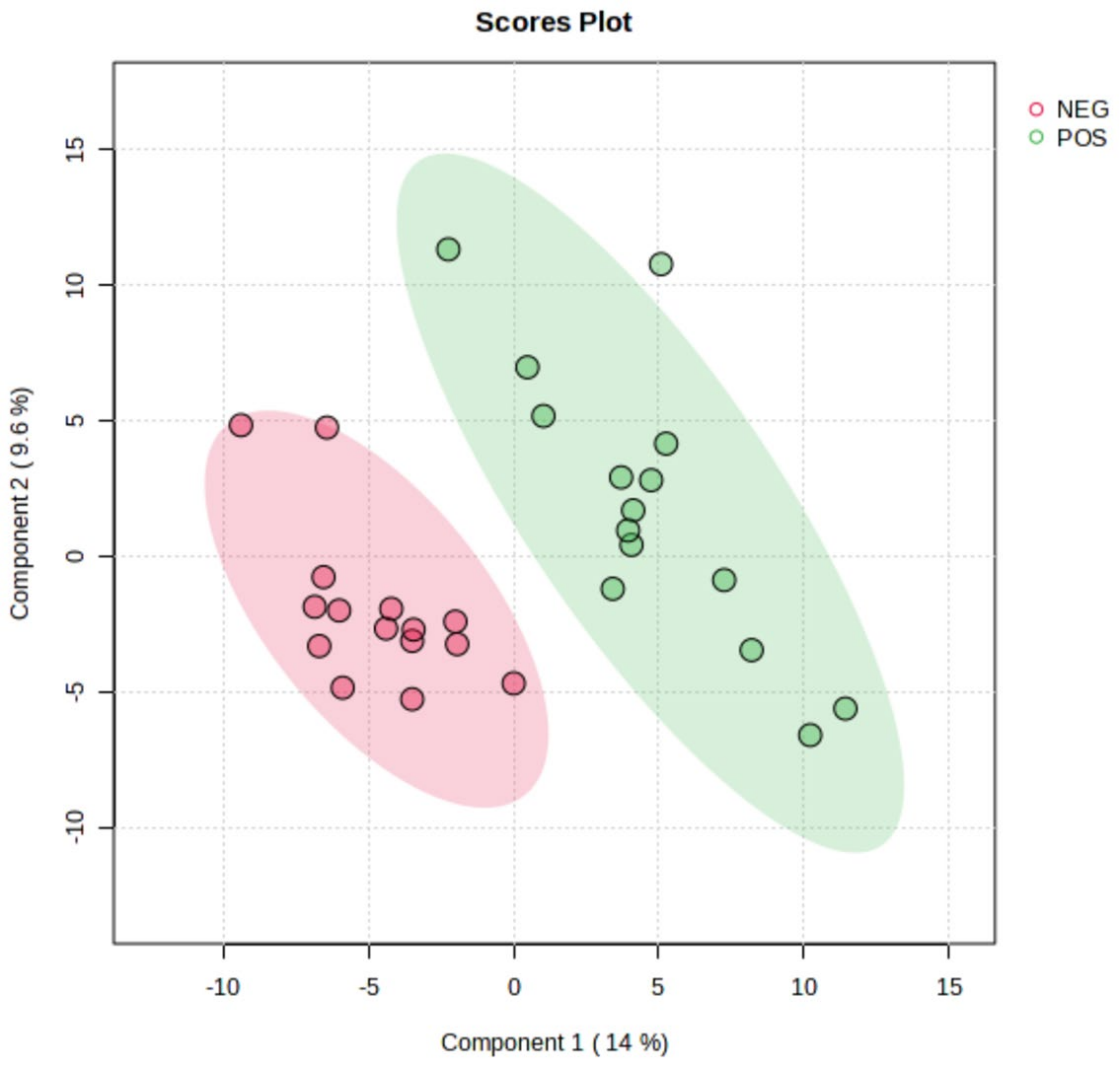
PLS-DA scores plot of the plasma proteome of beef steers with divergent residual body weight gain (RADG) phenotype.

**Figure 2:**
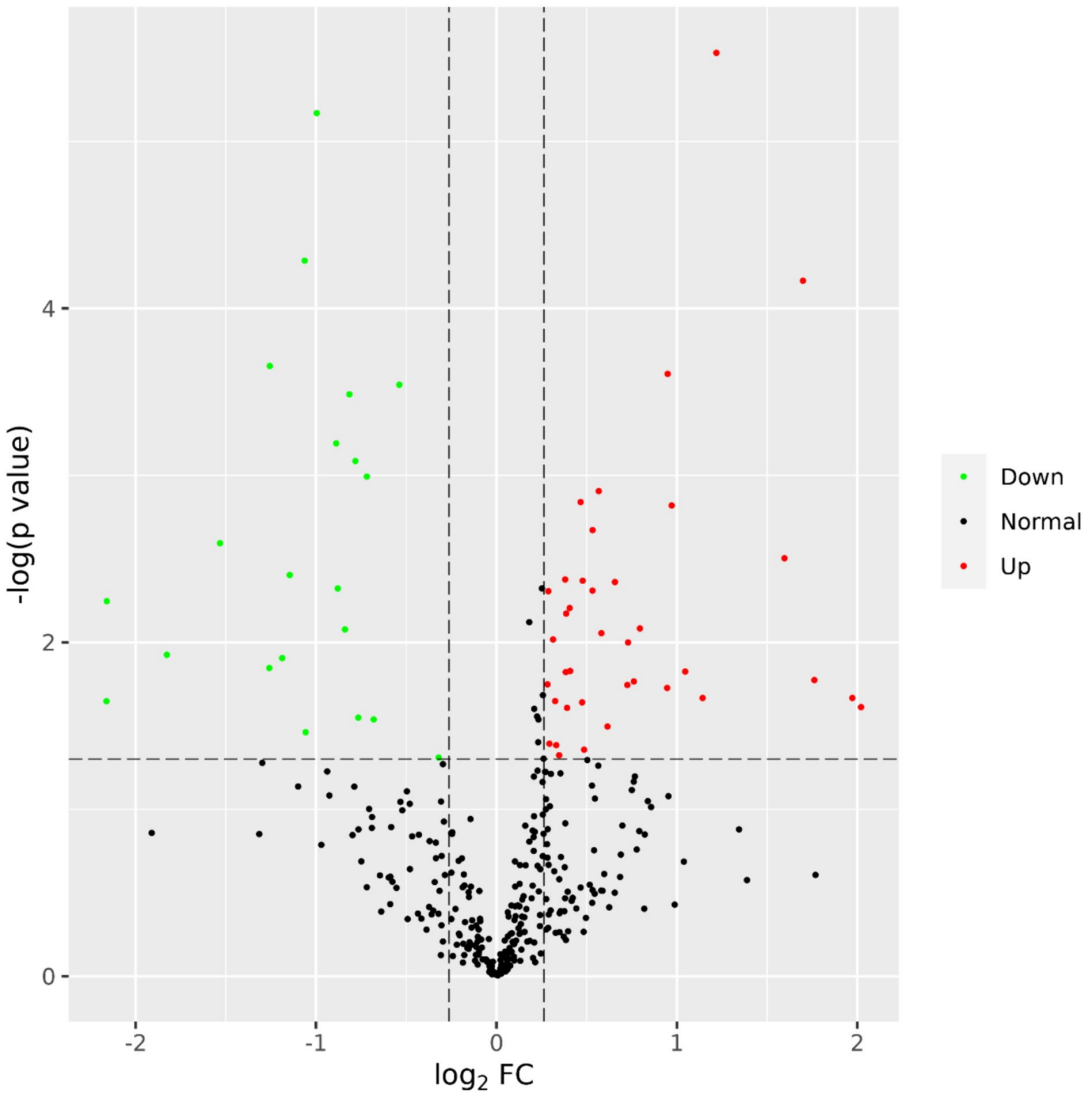
Volcano plot showing the differentially abundant proteins. Proteins with false discovery ratio ≤ 0.05 (red or green) are differentially increased (red dots) or reduced (green dots) in positive- residual body weight gain beef steers, relative to negative- residual body weight gain beef steers.

A total of 435 proteins were detected and identified (Supplementary Table 1). The PLS-DA score plot showed a clear separation between the two groups of beef steers using the first two principal components with 9.6 and 14% of explained variance (Figure 1), indicating differences in the plasma proteome profile between the two groups. A total of 59 differentially abundant proteins were detected between negative-RADG beef steers and positive-RADG beef steers (Figure 2). Plasma abundance of 38 proteins, including macrophage stimulating 1, peptidase D, plasma serine protease inhibitor, transthyretin, and adiponectin B were greater (FC ≥ 1.2, FDR ≤ 0.05) in positive-RADG beef steers while 21 proteins, including fibronectin, apolipoprotein F, MSH3 protein, and ALB protein, were greater (FC < 1.2, FDR ≤ 0.05) in negative-RADG beef steers.

### GO analysis of differentially abundant proteins

**Figure 3:**
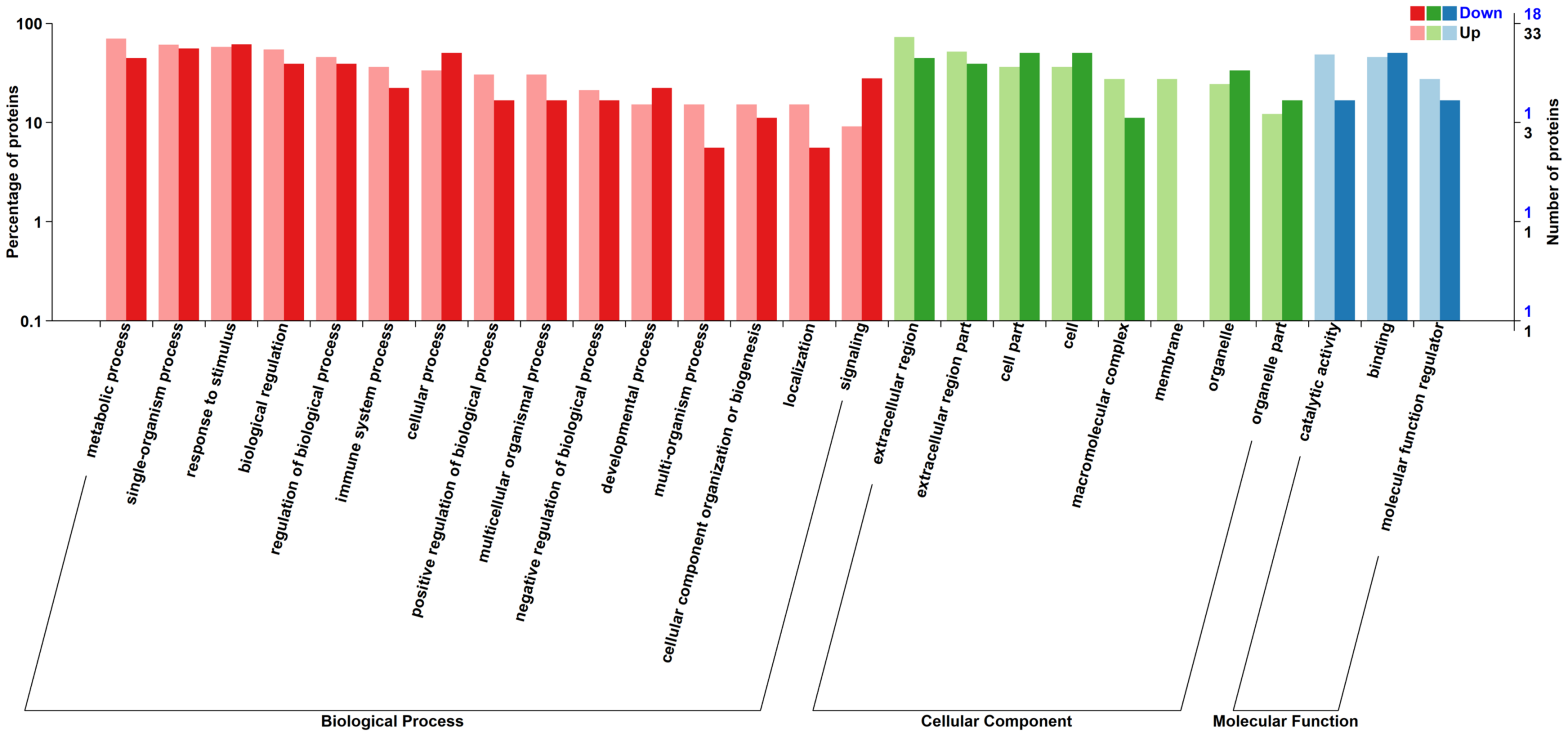
Distribution of differentially abundant proteins annotated in Gene Ontology level. *x*-axis displays Gene Ontology term; *y*-axis displays protein count. Up; Differentially abundant proteins associated with biological processes, cellular component, and molecular function greater in positive- residual body weight gain; Down; Differentially abundant proteins associated with biological processes, cellular component, and molecular function greater in negative- residual body weight gain beef steers.

The relative abundance of DAPs associated with metabolic processes, single-organism processes, biological regulation, multi-organism processes, biogenesis, and localization, catalytic activity and molecular function regulation were greater in positive-RADG beef steers (Figure 3). However, DAPs associated with processes such as signaling, developmental process, cellular process, organelle, and binding were lower in positive-RADG beef steers, compared to negative-RADG beef steers (Table 2).

**Table 2:** List of differentially abundant proteins in the plasma of beef steers with divergent residual body weight gain phenotype.

Protein ID	Protein names	Regulated
Q29437	“Primary amine oxidase, liver isozyme (EC 1.4.3.21) (Amine oxidase [copper-containing]) (Copper amine oxidase) (Serum amine oxidase) (SAO)”	Up
A0A3Q1LPQ0	Protein disulfide-isomerase (EC 5.3.4.1)	Up
A0A3Q1M2A8	Complement factor I	Up
Q1RMN8	“Immunoglobulin light chain, lambda gene cluster”	Up
B8Y9S9	Fibronectin	Down
Q0IID3	Guanylate binding protein 5	Up
A0A6B9SE94	Ig heavy chain variable region	Down
Q6T182	Sex hormone-binding globulin	Up
A0A6B9SE37	Ig lamda chain variable region	Up
A0A3Q1NGC5	Peptidase D	Up
P02070	Hemoglobin subunit beta (Beta-globin) (Hemoglobin beta chain) [Cleaved into: Spinorphin]	Down
Q862Q3	Beta-2-microglobulin	Up
A0A3Q1LP66	Plasma serine protease inhibitor	Up
G3MZF2	[histone H3]-lysine (4) N-methyltransferase (EC 2.1.1.364)	Down
A5PJH7	LOC788112 protein (Neutrophilic granule protein-like)	Down
A0A0A0MP92	Endopin 2	Up
A0A6B9SBR3	Ig lamda chain variable region	Up
A5D9E9	“Complement C1r (Complement component 1, r subcomponent)”	Up
A0A3Q1N2C2	Albumin domain-containing protein	Down
A0A6B9SF17	Ig heavy chain variable region	Up
Q2KIX7	Protein HP-25 homolog 1	Up
Q17QH1	APOF protein (Apolipoprotein F)	Down
Q9BGI3	Peroxiredoxin-2 (EC 1.11.1.24) (Thioredoxin-dependent peroxiredoxin 2)	Down
A0A3Q1LSX3	Macrophage stimulating 1	Up
F1MZ78	Leucine rich repeat and coiled coil centrosomal protein 1	Down
Q2KHW7	Regulator of G-protein signaling 10 (RGS10)	Down
B0JYQ0	ALB protein	Down
A0A6B9SF41	Ig lamda chain variable region	Down
Q9TT36	Thyroxine-binding globulin (Serpin A7) (T4-binding globulin)	Up
A0A3Q1LK49	Inter-alpha-trypsin inhibitor heavy chain 2	Up
E1BH06	Complement component 4A	Up
Q29423	CD44 antigen (Extracellular matrix receptor III) (ECMR-III) (GP90 lymphocyte homing/adhesion receptor) (HUTCH-I) (Hermes antigen) (Hyaluronate receptor) (Phagocytic glycoprotein 1) (PGP-1) (Phagocytic glycoprotein I) (PGP-I) (CD antigen CD44)	Up
P98140	Coagulation factor XII (EC 3.4.21.38) (Hageman factor) (HAF) [Cleaved into: Coagulation factor XIIa heavy chain; Coagulation factor XIIa light chain]	Up
O46375	Transthyretin (Prealbumin)	Up
P80109	Phosphatidylinositol-glycan-specific phospholipase D (PI-G PLD) (EC 3.1.4.50) (Glycoprotein phospholipase D) (Glycosyl-phosphatidylinositol-specific phospholipase D) (GPI-PLD) (GPI-specific phospholipase D)	Up
Q29RU4	Complement component C6	Up
E1BM23	Rho GTPase activating protein 5	Down
Q2KIU3	Protein HP-25 homolog 2	Up
Q2HJF0	Serotransferrin-like	Down
A0A140T851	“Protein C, inactivator of coagulation factors Va and VIIIa”	Up
E1BMJ0	Serpin family G member 1	Up
A0A3Q1MNU0	MIA SH3 domain ER export factor 3	Down
E1BH94	Peptidoglycan recognition protein 2	Up
A0A3Q1MSF6	Ig-like domain-containing protein	Down
G3N0V0	Ig-like domain-containing protein	Up
G5E5W1	Coagulation factor VIII	Up
Q2KIW4	Lecithin-cholesterol acyltransferase	Up
Q2KJ34	HMG box-containing protein 1 (HMG box transcription factor 1) (High mobility group box transcription factor 1)	Down
E1BCW0	HGF activator	Up
E1BL97	SRY-box transcription factor 13	Down
F1MH40	Ig-like domain-containing protein	Up
F1MFH5	Kelch like family member 42	Down
A0A3Q1LI44	Ig-like domain-containing protein	Down
Q2KJH6	Serpin H1 (Collagen-binding protein) (Colligin)	Up
A0A3B0IZF8	Adiponectin B (Complement C1q C chain)	Up
Q3SYW2	Complement C2 (EC 3.4.21.43) (C3/C5 convertase) [Cleaved into: Complement C2b fragment; Complement C2a fragment]	Up
A0A3Q1ME66	“ADP/ATP translocase (ADP, ATP carrier protein)”	Up
A0A3Q1NJ24	Carboxypeptidase B2	Up
A6QQE6	MSH3 protein	Down

Regulated: up, proteins upregulated in plasma proteome of positive- residual body weight gain beef steers; down, proteins downregulated in plasma proteome of positive- residual body weight gain phenotype beef steers.

### KOG analysis of differential abundant proteins

**Figure 4:**
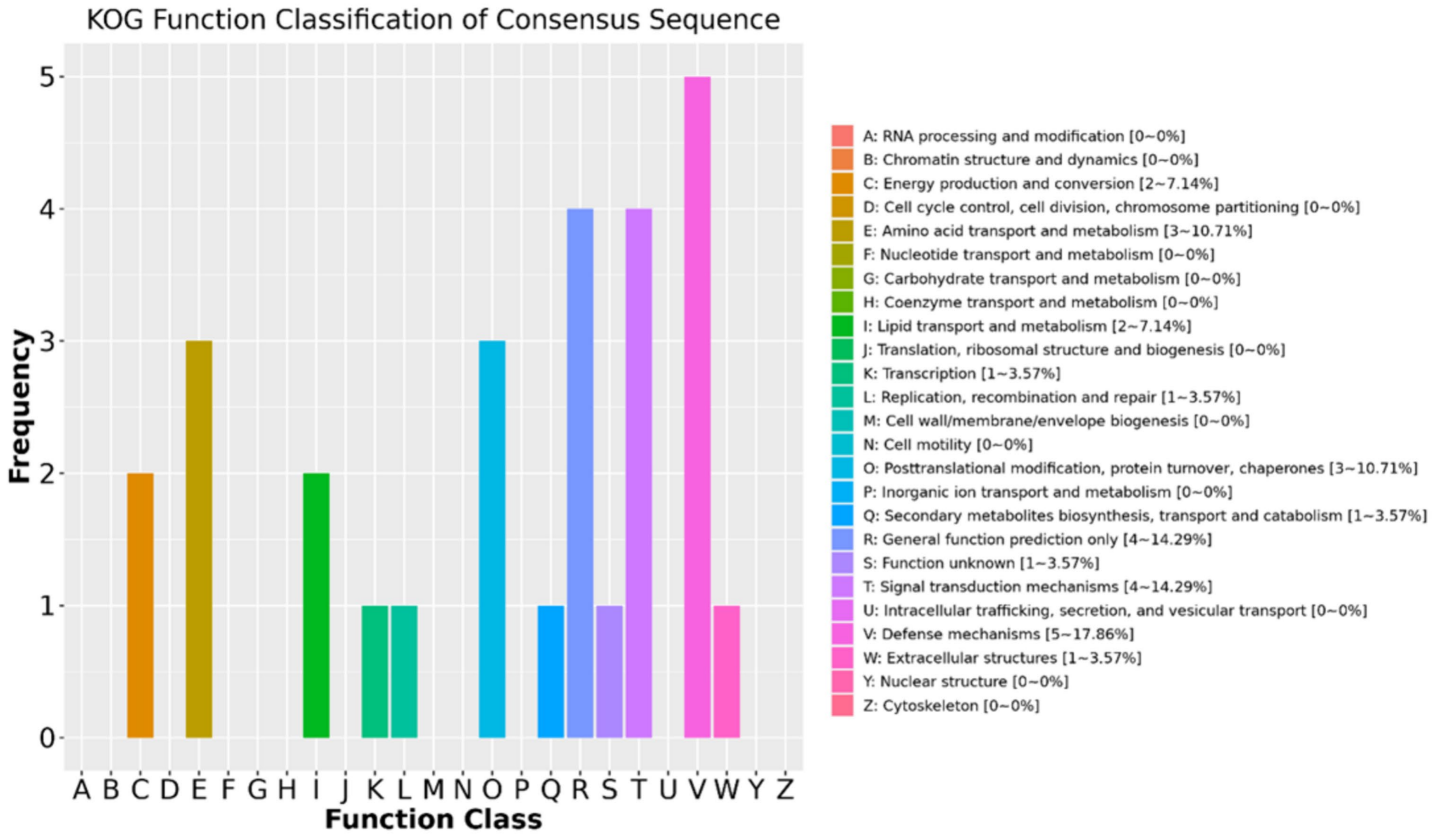
EuKaryotic Orthologous Group classification of the differentially abundant proteins. *x*-axis displays EuKaryotic Orthologous Group terms; *y*-axis displays protein count. Only 28 DAPs are annotated (24 of which are greater in positive-RADG beef steers) (see Supplementary Table 2).

The results of the KOG function classifications revealed that a total of 28 DAPs are annotated or classified in KOG and are associated with energy production and conversion, amino acid transport and metabolism, lipid transport and metabolism, transcription, replication recombination and repair, posttranslational modification, protein turnover, secondary metabolites biosynthesis, signal transduction mechanisms, defense mechanisms, and extracellular structures (Figure 4). Out of the 28 DAPs, 24 were greater in positive-RADG beef steers, compared to the negative-RADG beef steers (Supplementary Table 2).

## Discussion

Determining the metabolic mechanisms associated with feed efficiency, especially using accessible and non-invasive samples like blood, is crucial for the future of livestock production in terms of both profitability and animal welfare. This study aimed to identify metabolic pathways and key proteins associated with divergent RADG classification in beef steers. Using a plasma proteomics profiling approach, we identified and quantified 59 DAPs between the two groups of beef steers.

The number of DAPs involved in metabolic processes, single-organism processes, biological regulation, multi-organism processes, biogenesis, and localization were greater in positive-RADG beef steers. Livestock metabolic processes are fundamental for growth and overall health. These processes involve various biochemical pathways, hormonal regulation, and nutrient utilization. These processes contribute to the efficient breakdown and utilization of nutrients from the feed, ensuring that energy resources are effectively harnessed for growth and production. Upregulation of single-organism and multi-organism processes tailor physiological functions to maximize individual efficiency (18). Biogenesis processes contribute to the efficient formation of cellular structures and organelles. This ensures the proper functioning of cells, tissues, and organs, supporting overall health and performance (19). Biological regulation and localization ensure precise control over various physiological and metabolic pathways, ensuring the targeted delivery of nutrients to specific cellular compartments (20, 21). These processes also contribute to adaptive responses to environmental and nutritional challenges (22). Upregulation of these aforementioned processes in positive-RADG beef steers suggests that energy wastage is reduced, and resources are allocated to processes that positively impact feed efficiency (23).

In the molecular function category, DAPs associated with catalytic activity and molecular function regulation were notably higher in positive-RADG compared to negative-RADG beef steers. Catalytic activity plays a pivotal role in the digestion and absorption of nutrients, facilitating the efficient breakdown of complex molecules in the digestive system to allow for optimal processing of nutrients (24). Molecular function regulation involves the control of gene expression (25) and includes the control of hormonal signaling pathways (26). A balance in signaling could contribute to the coordinated regulation of physiological processes such as nutrient metabolism and energy utilization, which probably explain the increased feed efficiency of positive-RADG beef steers.

The DAPs involved in signaling, developmental processes, cellular processes, and binding were downregulated in positive-RADG beef steers compared to negative-RADG beef steers. Several studies have reported the association of genes related to immune signaling pathways with feed efficiency status in animals. For instance, (27) identified biological processes such as the immune signaling as potentially associated with RFI divergence in beef cattle. Similar findings were reported in several other studies (28–30). Signaling pathways play a crucial role in regulating various physiological processes related to immune function, feed intake, digestion, metabolism, and nutrient utilization in cattle, ultimately influencing feed efficiency (31, 32). However, upregulated signaling pathways and cellular processes often require additional energy for their activation and maintenance (33). Developmental processes such as growth and tissue remodeling require substantial energy resources (34); likewise, cellular processes often require higher energy expenditure for activities such as cell growth, maintenance, and repair (35). When these processes are upregulated, less energy is diverted toward supporting growth and development (34, 36). This increased energy demand could divert resources away from productive functions such as growth and production, ultimately reducing feed efficiency.

Using KOG analysis, DAPs associated with several pathways such as energy production and conversion, amino acid transport and metabolism, lipid transport and metabolism, transcription, replication recombination and repair, protein turnover, and defense mechanisms were greater in positive-RADG beef steers. Energy production and conversion plays a pivotal role in feed efficiency of animals, influencing both economic and environmental aspects of livestock production. This pathway plays a crucial role in converting dietary energy into usable forms that support various physiological functions, including growth, maintenance, and reproduction (37, 38). Previous studies have demonstrated that feed-efficient beef cattle show increased activity in energy production and conversion pathways, including glycolysis, the tricarboxylic acid (TCA) cycle, oxidative phosphorylation, and beta-oxidation (18, 39). Glycolysis is essential in glucose metabolism, converting glucose into pyruvate while generating ATP and NADH (40). Recent research by Xie et al. (41) and Elolimy et al. (42) further highlights the importance of glycolysis in feed efficiency, with both studies reporting elevated levels of glycolytic pathways in feed-efficient cattle. Additionally, the TCA cycle, which oxidizes acetyl-CoA from various nutrients to produce ATP, NADH, and FADH_2_, plays a crucial role in energy production (43). Previous studies reported an upregulation of the TCA cycle in more feed-efficient beef cattle, indicating their ability to efficiently extract energy from nutrients (42, 44).

The productivity of farm animals is significantly influenced by amino acid metabolism, as it plays integral roles in various biochemical and metabolic processes within animal cells. These processes, including growth, production, and reproduction, underscore the crucial contribution of amino acid metabolism to the overall efficiency and performance of farm animals (45, 46). Research by Elolimy et al. (42) observed that feed-efficient cattle exhibit upregulated metabolic pathways associated with both energy and amino acid metabolism in the rumen and skeletal muscle.

Lipids, including fatty acids, sterols, phospholipids, and triglycerides, serve as essential energy storage molecules in the body (47). Lipid metabolism involves the breakdown and synthesis of fatty acids, triglycerides, cholesterol, and ketone bodies, which play crucial roles in energy production, cell membrane structure, and hormone synthesis (47, 48). A recent study by Taiwo et al. (49) found that feed efficient beef steers exhibit upregulated molecular mechanisms related to fatty acid transport, fatty acid β-oxidation, and mitochondrial ATP synthesis. This suggests an enhanced metabolic capacity, enabling these beef cattle to maximize energy and nutrient utilization from their feed. In feed-efficient cattle, there is an upregulation of lipid metabolism in the liver, resulting in increased synthesis of lipoproteins. These lipoproteins play a crucial role in transporting lipids to various tissues, ensuring an effective delivery of lipids for energy production and other metabolic functions (50). The coordinated upregulation of lipid transport and metabolism pathways in feed-efficient cattle signifies a multifaceted approach to optimize energy utilization, contributing to their overall efficiency in nutrient utilization.

The upregulation of transcription, replication, recombination, and repair, along with posttranslational modification pathways in feed-efficient cattle, signifies intricate molecular adaptations contributing to increased metabolic efficiency and overall performance (51). Upregulated transcription ensures the streamlined synthesis of mRNA, thereby increasing the expression of genes potentially involved in nutrient metabolism and energy utilization. According to Tizioto et al. (52), this increased transcription of key genes associated with feed efficiency optimizes the processing of dietary nutrients and metabolic pathways.

For efficient feed utilization, a dynamic and responsive cellular environment is essential, necessitating the upregulation of DNA replication, recombination, and repair pathways (53). Feed-efficient cattle also exhibit increased activity in posttranslational modification pathways, including phosphorylation, acetylation, and ubiquitination. This heightened activity, as noted by Hunter (54), allows for swift and precise responses to changing nutritional conditions, further contributing to the adaptive capacity of feed-efficient cattle.

Protein turnover, encompassing both synthesis and degradation of proteins within cells (55), was upregulated in feed-efficient cattle. This increased turnover ensures the effective breakdown and synthesis of proteins, directing dietary amino acids toward essential proteins crucial for growth, metabolism, and overall physiological functions (56). Secondary metabolites, organic natural products synthesized by animals through enzymatic cascades, play diverse roles in biological functions such as defense, signaling, and adaptation (57). The upregulation of secondary metabolite biosynthesis in feed-efficient cattle contributes to improved nutrient absorption in the gastrointestinal tract, directly enhancing the utilization of dietary nutrients and positively influencing overall feed efficiency (58). Moreover, specific secondary metabolites, including alkaloids and flavonoids, known for their immunomodulatory properties, further enhance immune function in feed-efficient cattle (59).

Signal transduction mechanisms involve how cells receive and respond to signals from the extracellular environment or other cells (60). The upregulation of these pathways enhances the cattle’s ability to detect changes in nutrient availability, facilitating rapid and precise adjustments in physiological processes. This optimization of dietary nutrient utilization contributes to improved feed efficiency (61). Notably, research suggests that genes involved in neuronal signal transduction potentially influence feed efficiency by modulating feed intake in pigs (62). Extracellular structures, including the extracellular matrix, cell wall, or extracellular vesicles, surround or support cells (63). Upregulated extracellular structures play a role in maintaining the structural integrity of tissues and organs (64). This maintenance could enhance the overall health and well-being of feed-efficient cattle, allowing them to sustain their efficient conversion of feed resources.

Defense mechanisms safeguard cells from harmful agents or stimuli, such as pathogens, toxins, or stress (63). Therefore, the upregulation of defense mechanisms, particularly in the immune system, contributes to improved health in feed-efficient cattle. A robust immune response minimizes energy diversion toward combating infections, redirecting more resources to growth and production, thus positively impacting feed efficiency (65, 66).

## Conclusion

The results of this study revealed key metabolic pathways associated with divergent RADG phenotype in beef cattle. Our study provides valuable insights into the molecular underpinnings of feed efficiency in crossbred beef steers, offering a foundation for future research and practical applications in livestock production.

## Data availability statement

The original contributions presented in the study are publicly available. This data can be found here: https://massive.ucsd.edu, accession MassIVE MSV000095325.

## Ethics statement

The animal study was approved by the Institutional Animal Care and Use Committees of West Virginia. The study was conducted in accordance with the local legislation and institutional requirements.

## Author contributions

MI: Writing – review & editing, Writing – original draft, Visualization, Validation, Software, Methodology, Investigation, Formal analysis, Data curation, Conceptualization. GT: Writing – review & editing, Validation, Methodology, Investigation, Data curation. TS: Writing – review & editing, Visualization, Validation, Methodology, Investigation. AA: Writing – review & editing, Validation, Software, Methodology, Investigation. IO: Writing – review & editing, Visualization, Validation, Supervision, Software, Resources, Project administration, Methodology, Investigation, Funding acquisition, Formal analysis, Data curation, Conceptualization.

## Footnotes

^1^ www.ebi.ac.uk/GOA/

## Supplementary material

SUPPLEMENTARY TABLE 1 List of differentially abundant proteins detected and identified between negative-RADG and positive-RADG beef steers.
